# Effects of Exercise Rehabilitation on Blood Pressure of Patients after Myocardial Infarction

**Published:** 2010

**Authors:** Mehdi Kargarfard, Reza Rouzbehani, Fatema Basati

**Affiliations:** 1 Associate Professor, Department of Exercise Physiology, Faculty of Physical Education and Sport Sciences, University of Isfahan, Isfahan, Iran; 2 Specialist in Community Medicine, Isfahan University of Medical of Sciences, Isfahan, Iran; 3 Faculty of Physical Education and Sport Sciences, University of Isfahan, Isfahan, Iran

**Keywords:** Myocardial infarction, Exercise cardiac rehabilitation, Blood pressure, Functional capacity

## Abstract

**Objectives:**

Supervised exercise cardiac rehabilitation programs have been suggested to all patients specially patients with post-myocardial infarction (MI) for many years. However, limited information is available on the usefulness of exercise rehabilitation programs in chronic MI. The aim of this study was to evaluate the outcome of supervised exercise training on MI patients by measuring both physical and physiological factors.

**Methods:**

This was a semi-experimental randomized study. It included seventy two (35 cases, 37 controls) post-MI patients aged 40 to 67 years. They were randomly selected from those with MI based on WHO criteria who were referred to cardiac rehabilitation unit of Isfahan Shahid Chamran cardiovascular research center. After initial measurements including weight, height, functional capacity, diastolic blood pressure (DBP) and systolic blood pressure (SBP) in both resting and exercise states, patients were randomized into either the training group (n=35) or the control group (n =37). The training group had supervised aerobic training program, three times a week, with 60–70% of the maximal heart rate (HR) reserve for two months. After the training program was completed, all measurements were repeated in both groups. Data were analyzed using one-way analysis of variance (ANOVA) with repeated measures.

**Results:**

Patients in exercise group showed statistically significant improvement in resting HR (81.27±7.75 bpm vs. 74.17±10.11bpm, p≤0.001), resting SBP (125.92±9.30 mmHg vs. 123.54±6.82 mmHg, p≤0.01), SBP peak (150.22±7.12 mmHg vs. 133.54±6.82 mmHg, p≤0.001), HR peak (132.51±3.06 bpm vs. 142.00±3.14bpm, p≤0.001), and exercise capacity (8.49±1.18 METs vs. 9.42±1.19 METs, p≤0.01).

**Conclusions:**

The results from the study showed that a 2-month exercise rehabilitation program in post-MI patients is useful for improving both blood pressure and exercise capacity and should be encouraged more commonly.

## INTRODUCTION

Chronic essential hypertension is still the most common and major risk factor for developing cardiovascular disease.^1^,^2^ Clinical evidence suggests that in patients with blood pressure (BP) more than 180/100 mm Hg, the risk of developing coronary heart disease is about 5 folds higher than those with BP less than 120/80 mm Hg.^1^ Also, the Framingham Heart Study proved that participants with high BP had higher rates of CVD as compared to those with optimal BP levels.^3^ A meta-analysis encompassing 61 prospective studies underscored that the risk of cardiovascular death decreased linearly with decreasing systolic BP to less than 115 mmHg and diastolic BP to less than 75 mmHg.^4^ The decrease in BP observed with exercise training (ET) in chronic heart failure patients^4^,^5^ and healthy elders,^6^,^7^ is now a well-recognized phenomenon. ET is also recommended to lower BP in patients with CHF.^8^ The Treatment of Mild Hypertension Study (TOMHS) has shown that lifestyle modification including weight loss and increased physical activity contributes significantly to BP control.^9^ According to the 1964 World Health Organization definition, cardiac rehabilitation includes all actions undertaken to provide optimal physical, mental and social environment for the cardiac patient to let him or her regain maximal functional capacity in the society.^10^ Thus, cardiac rehabilitation should be multifaceted and comprehensive. Exercise rehabilitation is a major component of the comprehensive cardiac rehabilitation. During the last 30 years, a major breakthrough occurred in our thinking regarding the role of physical activity in patients with cardiovascular disease. Until 1960s, bed rest and/or major limitation of exercise were considered beneficial for the majority of patients. In contrast, moderate or even intense ET is currently used not only in the prevention of coronary heart disease, also as a therapeutic measure following myocardial infarction (MI), percutaneous coronary intervention, cardiac surgery, and permanent pacemaker or cardioverter-defibrillator implantations. For some years now, physical rehabilitation is also undertaken in patients with MIregardless of its etiology.^11^–^13^ Both cross-sectional and longitudinal studies have demonstrated a lower prevalence of hypertension in physically active people. Regular isotonic ET such as cycling or swimming produces modest reduction in BP in persons with mild to moderate hypertension.^14^ Regular aerobic ET has been shown to prevent or delay future coronary deaths in patients with coronary disease (known as secondary prevention). A meta-analysis of 22 randomized trials of exercise-based rehabilitation after MI in 4554 patients showed a 20% to 25% reduction both in overall mortality and in cardiovascular mortality.^14^,^15^ Similarly, a meta-analysis of 10 trials in 4347 patients showed that comprehensive rehabilitation had a beneficial effect on mortality, but not on the rate of recurrent MI.^16^ Most of the trials incorporated other lifestyle changes, such as smoking cessation and dietary modification, along with exercise. When the trials of exercise alone were analyzed separately, the results were directionally similar but not statistically significant and this may be because of the small number of such trials.^17^ Aerobic exercise is associated with an improvement in exercise tolerance. Exercise tolerance relates to exercise duration in a symptom-limited exercise test or more accurately as peak or maximal oxygen uptake (VO2 peak).^18^ Research suggested that aerobic exercise has been shown to contribute to modification of other coronary risk factors. After acute MI, aerobic exercise promotes a decrease in percentage body fat^19^, reduces BP^20^, decreases triglyceride levels, and increases HDL cholesterol.^21^ Two studies have also shown that ET combined with modification of risk factors, especially a low fat diet, resulted in regression of atherosclerotic disease. Evidence of these changes to atherosclerosis was based on serial angiograms.^22^,^23^ Therefore, the aim of this study was to determine the resting and exercise BP responses to regular aerobic ET in patients with MI.

## MATERIAL AND METHODS

### Patients

This was a randomized, controlled trial examining the effects of a 2-month, moderate-to-vigorous intensity, cardiovascular ET program on BP in patients following MI. After undergoing baseline testing, subjects were randomly assigned to exercise or control groups. The exercise groups participated in an 8-week supervised exercise program, whereas the control group was instructed to maintain their current life-styles. Written informed consent was obtained from all volunteers before participation, and the study was approved by the Cardiovascular Research Center, Isfahan, Iran. Eighty (40 cases and 40 controls) post-MI patients aged 40 to 67 years were randomly selected from those with MI diagnosed based on WHO criteria who were referred to cardiac rehabilitation unit of Isfahan Shahid Chamran cardiovascular research center. There were 24 males and 11 females (mean age, 57.71±4.93 years) in exercise group and 19 males and 18 females (mean age, 56.32 ± 5.98 years) in control group.

### Physical measurements

At the examination, trained study nurses measured participants’ weights and heights. Measurements were taken with subjects wearing light clothing and no shoes. Weight was measured using balance scales to within 100 g. Height was measured to the nearest 0.5 cm using a height rule taped vertically to a wall. BMI was computed as weight/height ^2^(kg/m ^2^).

### Blood pressure measurements

One week before the start of experimental study, subjects visited the laboratory in the morning after a 12-hour fast and having refrained from physical activity for 24 hours. BP at rest was measured after five minute rest from the right arm in a sitting position with the standard sphygmomanometer. The first phase of Korot-koff sounds was recognized as systolic BP and the fifth phase as diastolic BP. BP at rest was measured three times with one-minute pause in between. The mean of the first and the second BP measurements was used in the analyses based on the WHO MONICA protocol.^24^

### Exercise Tests

After the primary evaluation of patients by a cardiologist, they were asked to fill out the information forms. Then, they were permitted to enter the study. First, all patients were randomly assigned to exercise or control groups. The subjects then performed the symptom limited exercise test based on Naughton protocol on a motorized treadmill. The subject was made to run on a treadmill till exhaustion. At timed stages during the test, the speed (km/hr) and grade of slope (%) of the treadmill were increased. The Naughton protocol was selected due to its moderate intensity increase per stage.^25^ Briefly, during the Naughton protocol the subjects start to exercise at 2 mph, 0% gradient with the percentage gradient increasing by 3.5% every 2 min. A trained physician was present during all tests. BP and HR were measured every 2 min. The exercise test was terminated in the subjects if the target heart rate was achieved or they complained of fatigue. Exercise was also discontinued if there were abnormal changes like decrease in systolic BP of 10 mm Hg along with evidence of ischemia, abnormal ECG pattern like ST segment displacement, appearance of arrhythmias, an inappropriate BP response to increasing workloads, angina pectoris or exercise-induced bundle branch block or if subject complained of chest pain. After completion of the test, each subject was randomly assigned to either the exercise or control group. The ET group entered the supervised exercise program and the control subjects were asked to maintain their current behaviors throughout the 2-month study period. The aerobic ET consisted of 10 min warm up, 30 min treadmill walking/jogging, and 10 min cool down three times per week. Intensity was prescribed at 60–70% maximal HR reserve (60-70% maximal HR reserve: average about 6-7 metabolic equivalent (METs) while 1 MET = 3.5 mL O_2_/kg body weight/min). Exercise duration was gradually increased during the first 2 weeks until subjects were exercising for 45 to 60 minutes/session, which was then maintained throughout the remaining 2-month period. Resting BP was calculated by averaging the BP measurements following at least 10 min seated rest by the subject before each ET. To ensure compliance with the exercise prescription, exercise HR for each subject was continuously monitored by staff members. In addition, subject’s blood pressures were taken every 10 min during exercise to ensure the safety of the subject. After 2-week run-in period until BP stabilized by ET, the 8-week exercise program started. All subjects had data collection at baseline and in 8-weeks during the 2-month exercise program. At the end of the exercise rehabilitation program, patients from both the exercise and control groups were once put through measurements of the HR, systolic BP and exercise capacity variables.

### Statistical Analysis

All data are expressed as mean values (SD). Non-parametric tests were used to avoid potential errors from non-normal distribution of data. Paired student t-test was used to compare significant differences of the same variables within the group before and after the training period. Differences between the two groups were analyzed using analysis of variance with repeated measures. The Statistical Package for Social Sciences (SPSS) 17.0 for Windows was used for data analysis. P<0.05 was considered statistically significant.

## RESULTS

A total of 80 eligible post-MI patients were recruited from April 2009 to August 2009; 72 (50 male and 22 female) subjects completed the study within 8 weeks; 5 in the exercise group due to exacerbation of disease requiring a treatment change or hospital admission and 3 in the control group, because of family or work related difficulties were not able to complete the study and were not included in the data analysis. The analysis was therefore based on data from 72 patients. The mean age of the subjects was 40-67 years. Table 1 lists data (mean ± SD) for age, height, body mass, body mass index, resting HR and exercise capacity for all subjects before the 2-month study. There were no significant differences in all baseline characteristics of the physical and physiological measurements between exercise and control groups at baseline. At the initial exercise stress testing, before physical rehabilitation, there wasn’t any significant difference between the two groups in exercise capacity (METs), (Table 1).

**Table 1:** Baseline characteristics of post myocardial infarction patients in the control and exercise groups

Variables	Control group (n=37)	Exercise group (n=35)	P value
Age (years)	56.32±5.98	57.71±4.93	NS
Sex male (n)	23 (62%)	27 (77%)	NS
Height (cm)	167.41±7.58	168.74±7.36	NS
Weight (kg)	73.19±6.65	72.66±6.80	NS
BMI (kg/m2)	26.14±2.04	25.71±0.90	NS
Resting HR (bpm)	81.19±7.26	79.83±11.63	NS
Exercise capacity (METs)	8.37±1.25	8.23±1.15	NS

NS, non-significant; BMI, body mass index (kg/m2); HR, heart rate;bpm, beats per minute; METs, metabolic equivalents

**Table 2:** Comparisons ofblood pressure between the control and exercise groups at baseline and end of the study

Measurements	Control group		Exercise group	
	Baseline	End	Baseline	End
Resting HR (beats/min) ***	81.19±7.26	81.27±7.75^c^	79.83±11.63	74.17±10.11
Resting SBP (mmHg) **	123.57±10.18	125.92±9.30^b^	129.60±10.97	123.54±6.82
Resting DBP (mmHg) *	81.05±8.23	79.73±5.30^b^	81.43±8.44	78.80±4.34
HR peak (beats/min) ***	132.08±2.91	132.51±3.06^c^	130.93±4.65	142.00±3.14
SBP peak (mmHg) ***	147.46±7.82	150.22±7.12^c^	145.40±6.88	133.54±6.82
DBP peak (mmHg) *	83.95±8.47	82.59±6.19	86.43±8.44	84.80±4.34
METs**	8.37±1.25	8.49±1.18^c^	8.23±1.15	9.42±1.19

a p≤0.05

b p≤0.01

c p≤0.001 between the baseline and the end values.

* p≤0.05

** p≤0.01

*** p≤0.001 between the exercise and the control groups.

HR: heart rate, SBP: systolic blood pressure, DBP: diastolic blood pressure, METs: metabolic equivalents

In respect to table 1, functional capacity was 8.23±1.15 METs in exercise group and without significant difference from functional capacity in the control group,8.37±1.25 METs. Physical training was prescribed on the basis of initial exercise capacity and exercise tolerance, and the first part of rehabilitation was performed as a physical rehabilitation. At the end of phase of rehabilitation, the functional capacity was assessed again by exercise stress testing, with the same test protocol as initial exercise examination. (Table 2) lists mean ± SD values for systolic and diastolic BP for each group before and after the 2-month study. Patients in exercise group showed statistically significant improvement in resting HR, resting systolic and diastolic BP, systolic BP peak, HR peak and exercise capacity (Table 2). At the end of phase of rehabilitation, exercise capacity was increased in exercise group from 8.23±1.15METs to 9.42±1.19METs (p<0.001) and was also increased in control group, but non-significantly from 8.37±1.25METs to 8.49 ± 1.18 METs. At the end of phase of rehabilitation, at the same submaximal exercise level, the resting HR and systolic and diastolic BPs at rest were significantly reduced in exercise group, when compared with pre-training values (Table 2), while no changes were observed in the control groups. Also, in comparison with the baseline data, the exercise group showed significant higher scores on HR peak and exercise capacity following the 8-week exercisetraining program (Table 2). In contrast, no significant changes were observed in any of the physiological parameters in the control group (Table 2).One-way analysis of variance (ANOVA) with repeated measures revealed a statistically significant difference in exercise group compared to the control group in resting HR, resting systolic BP, systolic BP peak, HR peak and exercise capacity (main effect for time, p≤0.05, Table 2).ANOVA with repeated measures also revealed no significant differences between the exercise and the control groups in their physiological characteristics including diastolic BP at rest and diastolic BP peak (Table 2). However, there were significant differences between the two groups in resting HR, resting systolic BP, systolic BP peak, HR peak and exercise capacity (p≤0.001, p≤0.01, p≤0.001, p≤0.001, and p≤0.001, respectively Table 2).

## DISCUSSION

The aim of this study was to investigate the effects of 8 week exercise cardiac rehabilitation program on BPin post-MI patients. Results indicated a significant improvement in physical and physiological characteristics of patients with MI participating in exercise rehabilitation program in the experimental group in comparison with the control group. Although in past, patients with MI were advised to avoid exercise because of the risk of raised cardiologic impairment, nowadays, several studies have indicated the positive effects of physical training in some of the physical, physiological, psychological parameters and quality of life of MI patients.^26^,^27^ Also, several reports have previously shown that ET is safe and beneficial in MI patients.^26^–^30^ Isometric exercise such as weight lifting, pushing and pulling may increase BP and this kind of exercise may be harmful for patients with MI or congestive heart failure.^26^,^29^ Some studies have shown improvement in functional capacity after ET,^28^,^29^ but others have not.^31^–^33^ In our study, significant improvement was found in functional capacity after two months ET. In the present study, at the end of phase of rehabilitation, exercise capacity was significantly increased in exercise group from 8.23±1.15 METs to 9.42±1.19 METs. The increases in functional capacity of patients with MI were similar to those reported previously.^26^,^27^ The increases in various indices of physiological parameters appear to be related to the initial physical and physiological status, duration and intensity of exercise program and seem to show a real effect of “exercise rehabilitation program”. Therefore, gains in aerobic capacity were more demonstrable in most exercise group patients.

In this study also, results showed that exercise rehabilitation in the experimental group in comparison with the control group, had a positive effect on physical and physiological characteristics including resting HR, resting SBP, SBP peak and HR peak. The reductions in SBP in patients with MI were similar to those reported previously.^26^–^29^ However, the mechanisms associated with reductions in SBP following dynamic and isometric ET are poorly researched. Hegbom et al. (2006) and Yu et al. (2004) suggested that the observed reductions in BP following dynamicand isometric ET might be associated with baroreceptor resetting after repeated exposure to the established pressor response to dynamic and isometric contractions.^26^,^27^ A number of investigators have examined the effects of chronic ET on resting BP in hypertensive populations.^34^ It is generally accepted that the mechanisms underlying the sustained decrease in BP of hypertensive individuals after training include a decrease in the resting HR and a decrease in circulating catecholamines.^35^ Several mechanisms accounting for the antihypertensive effects of mild exercise have been proposed. It may arise from the decrease of plasma norepinephrine, the decrease of endogenous ouabain like substance, or the increase of prostaglandin E.^36^,^37^ The decrease of plasma renin activity (PRA) was also proposed to play a role.^36^,^37^ However, PRA and aldosterone levels of the subjects remained unchanged during our study period; this phenomenon is compatible with a previous study.^38^ Our previous study demonstrated that serum catecholamines did not reveal any significant changes (unpublished data). Other factors such as decreased sympathetic nervous system activity and increased sensitivity of the baroreceptor reflex after ET may exert beneficial influences on BP reduction.

## CONCLUSION

In conclusion, regular ET had significant effects on increasing exercise capacity and decreasing systolic BP but not diastolic BP. Therefore, supervised ET can be of value for these patients. The intensity of the training must always be customized for each patient, and it must be born in mind that these patients have poor prognosis and should be treated with extra care.
